# Bibliographical Notices

**Published:** 1844-06-29

**Authors:** 


### BIBLIOGRAPHICAL NOTICES.

*Traité analytique de la Digestion considérée particulièrement dans l'Homme et dans les Animiaux vertébrés.* Par M. Blondlot, Docteur en Médecine, Professeur (adjoint) de chimie a l'Ecole de Médecine de Nancy, &c., &c. 8vo. pp. 471, Paris, 1843.

We always look with some degree of suspicion on works that purport to give a view of the physiology or pathology of the digestive organs, inasmuch as all history shows them to have been topics on which many a one has endeavoured to ride into practice, and in several cases not unsuccessfully. The work before us is not one of this class. The author has studied the healthy play of the digestive organs; has committed the results of his investigations to writing; and has thought that his lucubrations might be of service to his professional brethren. He certainly has given a fair historical exposition of the notions that have been entertained on the subject, and if his work exhibit no novelty of any moment, it is not the less a good treatise on the subject. His view of the composition of the gastric juice differs from that generally given; and therefore we cite it.

"It is a clear and limpid fluid, somewhat, at first sight, like the urine. It has a slight odour *sui generis*. Its taste is feebly saline and sourish," [absolutely sour we should say from the examination of the fluid obtained from San Martin, the person on whom Dr. Beaumont's observations were made] "and it constantly reddens vegetable blues. Submitted to analysis, it furnishes bi-phosphate of lime, which communicates to it its acid reaction, conjointly with a little bi-phosphate of ammonia, chloride of sodium, mucus, and a peculiar organic principle, very changeable, which it has not, hitherto, been practicable to obtain in an isolated state, and which appears to be a kind of mucous matter, in a certain state of modification, which we cannot comprehend, and still less reproduce artificially. Whatever it may be, this matter acts in the manner of ferments, under the influence of a proper temperature, which appears to be restricted between 10° and 40° cent., for above this temperature it completely and irrevocably loses its virtue. In order that its specific action shall be put in play, it absolutely requires the concourse of some weak acid. As for the other elements of the gastric juice, none, with the exception of the water, appears to take part in this action, the cause of which must be attributed, to all appearance, to one of those influences of contact, which we designate under the generic expression of catalytic force." p. 456.

The whole process of digestion, M. Blondlot properly considers to be of a physical character.

---

*Cyclopædia of Practical Medicine.*—Parts IV. V. and VI.

We have heretofore announced the appearance of the three first *parts* of this publication. Since then, three more have appeared, completing the first volume, or one-fourth of the whole work. The favourable opinion which we expressed on former occasions from the specimens then before us, is in no degree lessened by a further acquaintance with its scope and execution. Contrary to what has been urged elsewhere, we are decidedly of opinion that one great merit of this work consists in its being the production of a number of distinguished authors, writing their several parts simultaneously. Apart from the great advantage of the different monographs, of which it consists, emanating from gentlemen who have paid special attention to the subjects on which they write, by this arrangement we are put into early possession of the entire work. If confided to a single pen, years would necessarily elapse before it could be completed, if completed at all. During that time, such is the progressive character of our science, to say nothing of the inconveniences and danger of mishaps from so much delay, the *first* and the *last* of the work would scarcely belong to the same era.

